# Campbell title registrations to date

**DOI:** 10.1002/cl2.1335

**Published:** 2023-06-14

Details of new titles for systematic reviews or evidence and gap maps that have been accepted by the Editor of a Campbell Coordinating Group are published in each issue of the journal. If you would like to receive a copy of the approved title registration form, please send an email to the Managing Editor of the relevant Coordinating Group.

## CHILDREN AND YOUNG PERSONS WELLBEING

The FRIENDS preventive programme for reducing anxiety symptoms in children and adolescents: A systematic review

Trine Filges

1 March 2023

Parenting interventions to prevent out‐of‐home placement of children: A systematic review

Nina Thorup Dalgaard

21 April 2023

## CLIMATE SOLUTIONS

Interventions and infrastructure to mitigate human health impacts of extreme heat events in temperate climate zones: An evidence and gap map

Rachel Jansen

17 April 2023

Crime and Justice

Gambling courts for reducing problem gambling and recidivism in the criminal justice system: A mixed‐methods systematic review

Aino Suomi, Lorana Bartels, Meredith Rossner, Amanda Roberts

9 May 2023

Reducing community violence: A meta‐review of what works

David Wilson

4 May 2023

Interventions to respond to violence against women in high‐income countries: An evidence and gap map

Lorelei Hine

17 April 2023

Co‐responding police‐mental health programs and the impact on justice and social service outcomes: A systematic review

Matthew J. Teti, Catherine Clare Strange, Jordan M. Hyatt, Robert J. Kane

1 March 2023

## EDUCATION

Assessing the outcomes and impact of professional doctorate programmes in health and social care on the individual, their profession, their employing organisation and wider society: A systematic review

Hazel Chapman

26 April 2023

Identifying instructional design strategies for improving differentiated instruction in K‐12 education in China: A systematic review

Haizhi Fu

3 March 2023

## SOCIAL WELFARE

Abortion and women's mental health: A systematic review

Julia Littell

9 May 2023

Interventions to support violence against women victim‐survivors in high‐income countries to heal and recover: An evidence and gap map

Elizabeth Watt

9 May 2023

Medical‐financial partnerships for improving financial and medical outcome for lower‐income Americans: A systematic review

Julie Birkenmaier

1 May 2023

